# Synergy of Subgroup J Avian Leukosis Virus and Chicken Infectious Anemia Virus Enhances the Pathogenicity in Chickens

**DOI:** 10.3390/microorganisms12040740

**Published:** 2024-04-05

**Authors:** Huijuan Xu, Wenxue Li, Yu Nie, Sheng Chen, Hongxin Li, Xinheng Zhang, Qingmei Xie, Weiguo Chen

**Affiliations:** 1 State Key Laboratory of Swine and Poultry Breeding Industry, College of Animal Science, South China Agricultural University, Guangzhou 510642, China; sunnyxu20181@outlook.com (H.X.); 18846921030@163.com (W.L.); thcscau@163.com (Y.N.); chens@stu.scau.edu.cn (S.C.); hxli@scau.edu.cn (H.L.); xhzhang@scau.edu.cn (X.Z.); 2 South China Collaborative Innovation Center for Poultry Disease Control and Product Safety, Guangzhou 510642, China; 3 Key Laboratory of Animal Health Aquaculture and Environmental Control, Guangzhou 510642, China; 4 Heyuan Branch, Guangdong Laboratory of Lingnan Modern Agricultural Science and Technology, Heyuan 517001, China

**Keywords:** subgroup J avian leukosis virus, chicken infectious anemia virus, synergistic pathogenicity

## Abstract

Subgroup J avian leukemia virus (ALV-J) and chicken infectious anemia virus (CIAV) are widely acknowledged as significant immunosuppressive pathogens that commonly co-infect chickens, causing substantial economic losses in the poultry industry. However, whether co-infection of ALV-J and CIAV have synergistic pathogenicity remains uncertain. To explore their synergistic pathogenesis, we established a co-infection model of ALV-J and CIAV in HD11 cells and specific-pathogen-free (SPF) chickens. We discovered that ALV-J and CIAV can synergistically promote the secretion of IL-6, IL-10, IFN-α, and IFN-γ and apoptosis in HD11 cells. *In vivo*, compared to the ALV-J and CIAV mono-infected group, the mortality increased significantly by 27% (20 to 47%) and 14% (33 to 47%) in the co-infected group, respectively. We also discovered that ALV-J and CIAV synergistically inhibited weight gain and exhibited more severe organ damage in co-infected chickens. Furthermore, we found that CIAV can promote the replication of ALV-J in HD11 cells and significantly enhance ALV-J viral load in blood and tissues of co-infected chickens, but ALV-J cannot promote the replication of CIAV. Moreover, by measuring the immune organ indexes and proportions of blood CD3^+^CD4^+^ and CD3^+^CD8^+^ lymphocytes, more serious instances of immunosuppression were observed in ALV-J and CIAV co-infected chickens than in mono-infected chickens. Taken together, our findings demonstrate that ALV-J and CIAV synergistically enhance pathogenicity and immunosuppression.

## 1. Introduction

Immunosuppressive viral diseases cause increased susceptibility to secondary infections and sub-optimal response to vaccinations in chickens, posing a significant threat to the global poultry industry [1,2]. Subgroup J avian leukosis virus (ALV-J) and chicken infectious anemia virus (CIAV) are classical immunosuppressive viral pathogens in chickens [3]. ALV-J infection mainly causes tumor mortality, growth retardation, and serious immunosuppression in chickens [4]. Clinical manifestations of ALV-J infection include hepatomegaly and splenomegaly [5]. Additionally, research studies have indicated a tendency of ALV-J to target the bone marrow, the central immune organ. Early infection with ALV-J triggers lymphocyte apoptosis, leading to dysplasia in the thymus and Fabricius [6].

CIAV infection generally results in host aplastic anemia, systemic lymphoid tissue atrophy and severe immunosuppression [7]. The illness predominantly impacts chicks aged 1 to 3 weeks, and is characterized by symptoms of intramuscular bleeding, thymus atrophy, weight loss, and myelodysplasia, whereas adult chickens typically exhibit subclinical infection [8,9,10,11]. Owing to its nonspecific symptoms, this disease is frequently overlooked. Nonetheless, it is noteworthy that outbreaks of CIAV infection have been documented in 20-week-old laying hens, especially in instances of co-infection where immunosuppressive factors could be significant [12]. *In vitro* infection and proliferation experiments revealed that CIAV replicated in chicken embryos and lymphoblastic cell lines, but not in chicken embryo fibroblasts (CEFs), chicken kidney cells, or other primary cell types [13]. Chicken macrophages are crucial immune cells when combating diverse viral infections and serve as susceptible targets for both ALV-J and CIAV [14].

A growing body of evidence indicates that co-infection of immunosuppressive viruses is ubiquitous in poultry, resulting in more severe pathogenic effects [15]. For instance, our recent study has demonstrated that the synergistic viral replication of ALV-J and infectious bursal disease virus (IBDV) induces more severe immunosuppression in chickens and enhanced the pathogenicity as a result [16]. In addition, the synergistic pathogenic effect of ALV-J and avian reticuloendotheliosis virus (REV) co-infection leads to more serious growth retardation, immunosuppression, and secondary E. coli infection in broiler chickens [17]. Previous studies have also indicated that the co-infection of ALV-J with Marek’s disease virus (MDV) or *E. tenella* caused more severe pathogenicity, growth inhibition, mortality, and immunosuppression than mono-infections [18,19]. Additionally, CIAV chickens co-infected with avian reovirus or Gyrovirus homsa 1 have exhibited synergism in promoting viral replication, immunosuppression, and more serious tissue damage [20,21].

Since ALV-J and CIAV are mainly transmitted vertically through eggs, and could be transmitted horizontally through other media, too, these two viruses are highly contagious in chickens [22,23], leading to the prevalence of CIAV and ALV-J infections [24,25,26,27,28], which are responsible for huge economic losses to the poultry industry worldwide. In addition to the ALV-J or CIAV infection alone, several recent reports have described clinical cases of co-infection of ALV-J and CIAV in Chinese chicken flocks [29]. However, the synergy of ALV-J and CIAV remains inadequately elucidated. In this study, we established a co-infection cellular model and animal model of ALV-J and CIAV to investigate the synergistic pathogenicity *in vitro* and *in vivo*.

## 2. Materials and Methods

### 2.1. Viruses, Cells, and Animals

The SCAU-HN06 strain of ALV-J and the GD-101 strain of CIAV were stored in our laboratory. The 50% tissue culture infectious dose (TCID_50_) of the SCAU-HN06 strain of ALV-J was titrated by limiting dilution in the DF-1 culture. The GD-101 strain of CIAV was propagated in the MSB1 cells and titrated as previously described [30]. The chicken macrophage-like cells HD11 (stored in our laboratory), as well as DF-1 and MSB1 cells were cultured in Dulbecco’s modified Eagle’s medium (Life Technologies, Carlsbad, CA, USA) and supplemented with 10% fetal bovine serum (Life Technologies) and 1% penicillin–streptomycin (Life Technologies) at 37 °C in a 5% CO_2_ incubator. One-day-old SPF chicks (White Leghorn), including both hen chicks and cock chicks, were purchased from Xinxing Dahuanong Poultry Eggs Co., Ltd. (Yunfu, China).

### 2.2. Real-Time Quantitative PCR (qPCR) for the Detection of CIAV

Viral DNA was extracted from HD11 cells or tissues by using the HiPure Tissue DNA Mini Kit (Magen, Guangzhou, China) according to the manufacturer’s instructions. The primers (forward: 5′-GGACCATCAACGGTGTTCAGG-3′ and reverse: 5′-GTCGCAGGATCGCTTCTTCGA-3′) for detecting the VP3 of CIAV were designed based on the sequence of the GD-101 strain of CIAV. The viral load of CIAV was detected by qPCR with SYBR Green qPCR Master Mix (Glpbio, Montclair, CA, USA). The samples were amplified and analyzed in a Bio-Rad Laboratories CFX96 real-time fluorescence quantitative PCR apparatus (Shanghai, China) with the following program: 95 °C for 30 s, 40 cycles of 95 °C for 5 s, 60 °C for 35 s. The results were analyzed using the 2^−∆∆CT^ method.

### 2.3. Cell Infection and Sampling Design

**Figure 1:**
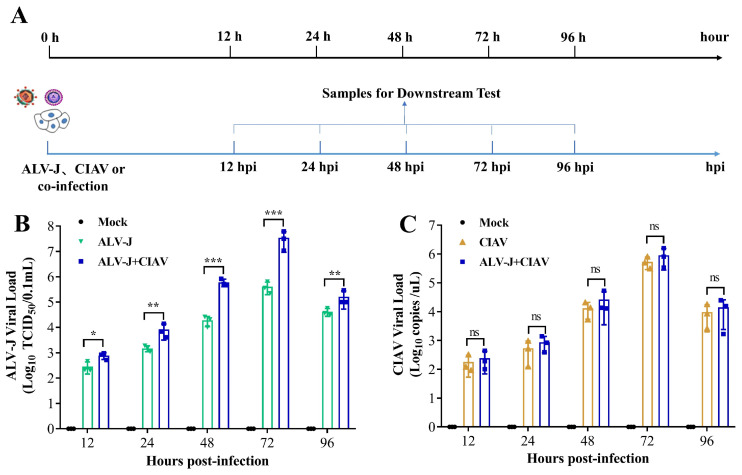
A time course diagram showing that *in vitro* and CIAV can increase the replication of ALV-J. (**A**) Time course of simultaneous co-infection of ALV-J and CIAV in HD11 cells. The cultured monolayer cells were infected with ALV-J and CIAV. At 12, 24, 48,72, and 96 hpi, cell samples were collected for downstream testing. All the experiments were performed independently at least three times (hpi, hours post infection) (**B**,**C**). ALV-J and CIAV virus titers in HD11 cells were determined from 12 to 96 hpi by qPCR. Data were expressed as mean ± SE and analyzed by a one-way ANOVA Test. These experiments were performed independently at least three times with similar results. *, *p* < 0.05; **, *p* < 0.01; ***, *p* < 0.001; ns—not significant.

HD11 cells were infected with ALV-J, CIAV, and ALV-J + CIAV in 12-well plates at a multiplicity of infection (MOI) of 1 of ALV-J or 1 × 10^6^ copy numbers of CIAV per well. The cells treated with equal amounts of phosphate-buffered saline (PBS) were used as mock-infected controls. The designated time course of infection and sampling is shown in Figure 1A.

### 2.4. Testing Indices of Cell Infection Experiments

At 12, 24, 48, 72, and 96 h post-infection (hpi), HD11 cell supernatants were collected to test the viral load of ALV-J or CIAV and the concentrations of cytokines. The ALV-J and CIAV viral load were quantified by using the published qPCR method [16] and the qPCR method as described above, respectively. The concentrations of IL-6, IL-10, IFN-α, and IFN-γ in the cell supernatants were measured through ELISA as previously described [16]. At 12, 24, and 96 hpi, HD11 cells were collected to test the apoptosis using a Dead Cell Apoptosis Kit with annexin V-fluorescein isothiocyanate and propidium iodide (Beyotime). The percentage of apoptotic cells was quantitated by using a fluorescence-activated cell-sorting (FACS) BD AccuriC6 cell sorter (Becton, Dickinson and Company, Franklin Lakes, NJ, USA).

### 2.5. Animal Infection Experiments

A total of 160 one-day-old SPF chickens were randomly divided into 4 groups (40 chicks per group) and were housed in four separate negative-pressure-filtered air isolators. ALV-J mono-infected chickens were inoculated with 10^4.5^ TCID50 of the ALV-J strain SCAU-HN06 in 0.2 mL PBS through intraabdominal injection. CIAV mono-infected chickens were inoculated with 1 × 10^6^ copy numbers of the CIAV strain GD-101 in 0.2 mL PBS by leg muscle injection. Co-infected chickens were inoculated with 10^4.5^ TCID50 of the ALV-J and 1 × 10^6^ copy numbers of the CIAV in 0.2 mL PBS. Mock-infected chickens were inoculated with 0.2 mL PBS. All animal experiments were performed following the guidelines of the South China Agricultural University Animal Care and Use Committee (permit no. SCAU 2021b020). At the end of the experiment, all the chickens were euthanized with CO^2^. 

### 2.6. Testing Indices of Animal Experiments

The chickens in each group were clinically inspected daily after infection, and their weights and mortality were recorded each week throughout the experimental period. Venous blood samples from three chickens randomly selected from each group were collected in vacuum tubes at one-week intervals and then utilized for the ALV-J and CIAV viral load tests by qPCR. Three chickens selected randomly from each group were euthanized humanely and necropsied at 7, 21, and 42 days post-infection (dpi), and tissues from the spleen, thymus, Fabricius, liver, and kidney were collected to determine the viral load of ALV-J or CIAV in different organs by qPCR, as described above. At 21 and 42 dpi, samples of the immune organs, including the spleen, thymus, and Fabricius, were excised from 6 chickens in each group and weighed. The immune organ indices were calculated as organ weight (mg)/body weight (g) × 100%. At 21 dpi, 3 chickens selected randomly from each group were euthanized humanely, and necropsy tissue samples from the spleen, thymus, bursa of the Fabricius, liver, and kidney were collected for histopathological examination. At 7 dpi, anticoagulant-treated blood samples were collected from three chicks in each group and then utilized for analysis of peripheral blood lymphocyte subsets by fluorescence-activated cell-sorting (FACS) as previously described [16].

### 2.7. Statistical Analysis

The statistical analyses in this study were performed using the GraphPad Prism version 8.0 (San Diego, CA, USA). Survival curves between the two groups were compared using a log–rank test (Mantel–Cox). Comparisons of the viral load, cytokines expression, immune organ indices, and peripheral blood lymphocyte subsets data between two groups at different time points or tissues were performed using multiple *t*-tests (such as the Holm–Sidak method) and one-way analysis of variance (ANOVA). Different lowercase letters indicate significant differences between different groups. Differences were considered statistically significant at *p* < 0.05. *p* values of less than 0.05, 0.01, and 0.001 are indicated with *, ** and ***, respectively.

## 3. Results

### 3.1. CIAV Can Increase the Replication of ALV-J In Vitro

To investigate the synergistic effects of the replication of ALV-J and CIAV *in vitro*, HD11 cells were infected with phosphate buffer (Mock), ALV-J, CIAV, and both viruses (ALV-J + CIAV), respectively. The time course of infection and sampling is shown in Figure 1A. As expected, the ALV-J RNA could not be detected in CIAV mono-infected and mock-infected HD11 cells; on the other hand, the ALV-J viral load of co-infected HD11 cells was higher than that of ALV-J mono-infected HD11 cells at 12 hpi, and this difference was extremely significant from 24 hpi to 96 hpi (Figure 1B). However, the CIAV copy number in CIAV mono-infected and co-infected HD11 cells did not differ significantly in any time detected with regard to the stage of infection (Figure 1C). These results demonstrate that CIAV can significantly promote the ALV-J replication in chicken macrophage cells but ALV-J cannot enhance the CIAV replication *in vitro*.

### 3.2. ALV-J and CIAV Synergistically Induce Inflammatory Mediator Secretion and Apoptosis 

**Figure 2:**
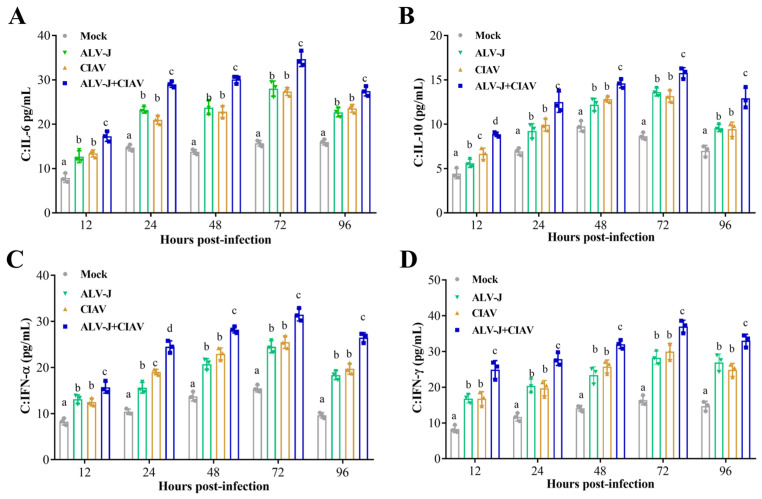
ALV-J and CIAV synergistically induce inflammatory mediator secretion *in vitro*. (**A**) IL-6 (**B**) IL-10 (**C**) IFN-α and (**D**) IFN-γ in cells at 12, 24, 48, 72 and 96 dpi were detected by enzyme-linked immunosorbent assay (ELISA). Data were expressed as mean ± SE and analyzed by a one-way ANOVA test. Different lowercase letters indicate significant differences between different groups.

To explore whether the two viruses, ALV-J and CIAV, could synergically promote the secretion of cytokines, we tested the dynamic changes in IL-6, IL-10, IFN-α and IFN-γ secretion in HD11 cells by ELISA. The results showed that the levels of IL-6, IL-10, IFN-α, and IFN-γ (from 12 hpi to 96 hpi) in the co-infection cells were significantly higher compared to the ALV-J and CIAV mono-infected cells or the controls (Figure 2). The data indicated that ALV-J and CIAV synergistically induce the secretion of inflammatory mediators *in vitro*.

**Figure 3:**
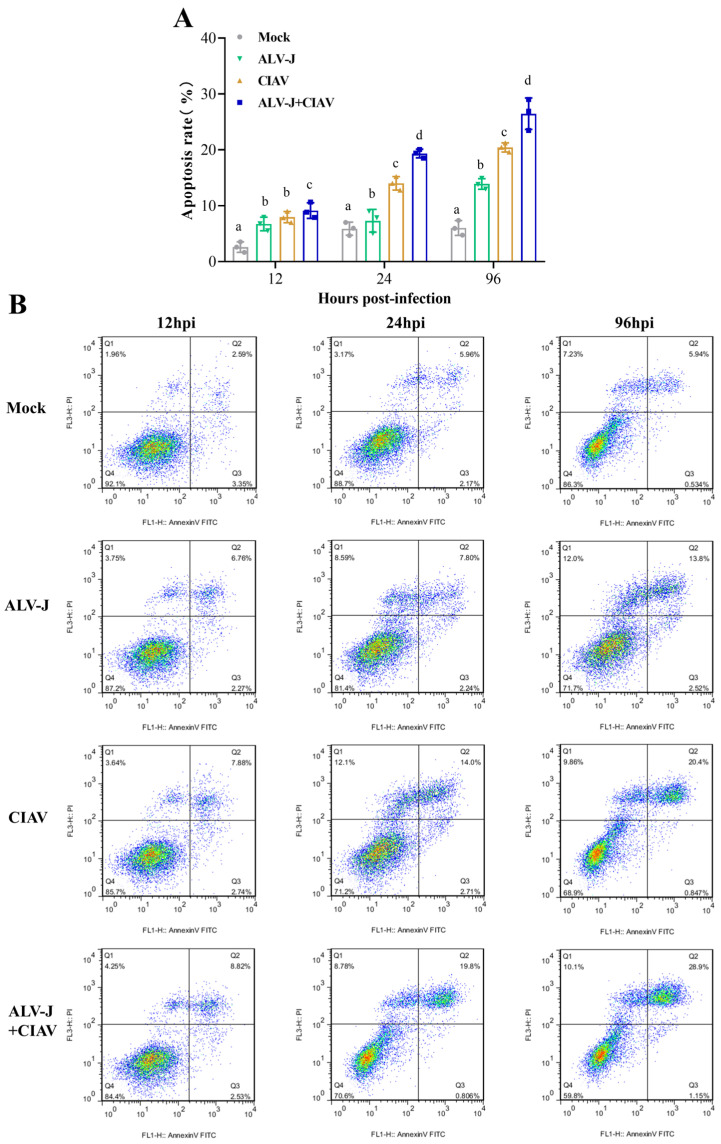
ALV-J and CIAV synergistically induced apoptosis of HD11 cells. (**A**). Statistics of cell apoptosis rate in each group. Data were expressed as mean ± SE and analyzed by a one-way ANOVA test. Different lowercase letters indicate significant differences between different groups. (**B**). Scatter plots of apoptosis detected by flow cytometry in each group.

Next, we collected HD11 cells to evaluate the effect of ALV-J or CIAV mono-infection and co-infection on apoptosis. As shown in Figure 3, there was no significant difference in the apoptosis rate between ALV-J and CIAV mono-infected cells, but both of them had significantly higher rates than that of mock-infected cells at 12 hpi. Notably, the apoptosis rate of the co-infected cells was significantly higher than that of mono-infected cells at 12 hpi. The apoptosis rate of CIAV mono-infected cells was significantly higher than that of ALV-J mono-infected or mock-infected cells at 24 hpi and 96 hpi, while the ALV-J and CIAV co-infected cells had significantly higher apoptosis rates than ALV-J or CIAV mono-infected cells at 24 hpi and 96 hpi. These results revealed that ALV-J and CIAV indeed synergistically promote apoptosis and cause more serious harm to HD11cells.

### 3.3. ALV-J and CIAV Synergistically Increase the Pathogenicity in SPF Chickens

**Figure 4:**
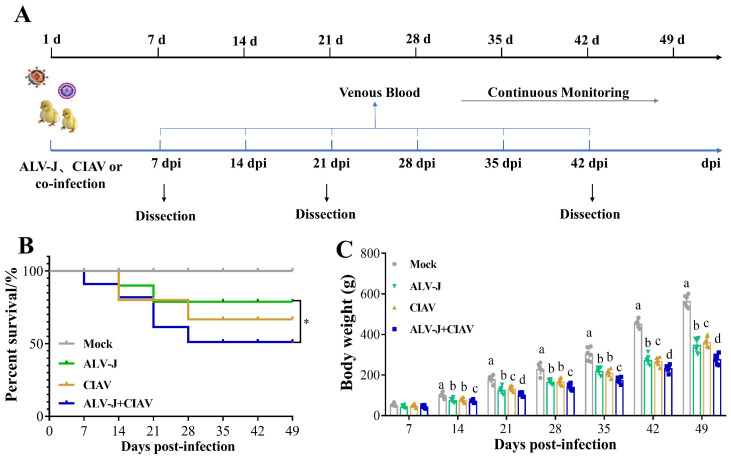
ALV-J and CIAV synergistically increase the pathogenicity in SPF chickens. (**A**). Time course of simultaneous co-infection of ALV-J and CIAV in SPF chickens. After infection, venous blood from 3 chickens was collected from each group weekly to detect the virus load. At 21 and 42 dpi, samples of the immune organs, including the thymus, Fabricius, and spleen, from 3 chickens in each group were excised and weighed. Weight loss and mortality were continuously monitored throughout the experimental period. (dpi—days post infection). (**B**). Survival curves for each group. (**C**). Body weight of SPF chickens for each group from day 1 to day 49. Data were expressed as mean ± SE and analyzed by one-way ANOVA Test. Different lowercase letters indicate significant differences between different groups. These experiments were performed independently at least three times with similar results. *, *p* < 0.05; ns—not significant.

To further understand the co-pathogenicity of ALV-J and CIAV *in vivo*, we performed animal infection experiments on one-day-old SPF chicks and the experimental process is illustrated in Figure 4A. As shown by the survival curves in Figure 4B, none of the uninfected chickens showed clinical symptoms and mortality, while the overall mortality of co-infected chickens was significantly higher than that of mono-infected chickens. The ALV-J and CIAV mono-infected chickens had an overall mortality of 20% and 33%, respectively, while the co-infected chickens had 47%. The average body weight of ALV-J and CIAV co-infected chickens was significantly lower than that of ALV-J and CIAV mono-infected chickens or uninfected chickens from 14 dpi to 49 dpi. The average body weight of co-infected, ALV-J, CIAV mono-infected, and uninfected chickens was 267.5 g, 322.8 g, 359.3 g, and 506.6 g at 49 dpi, respectively (Figure 4C). 

**Figure 5:**
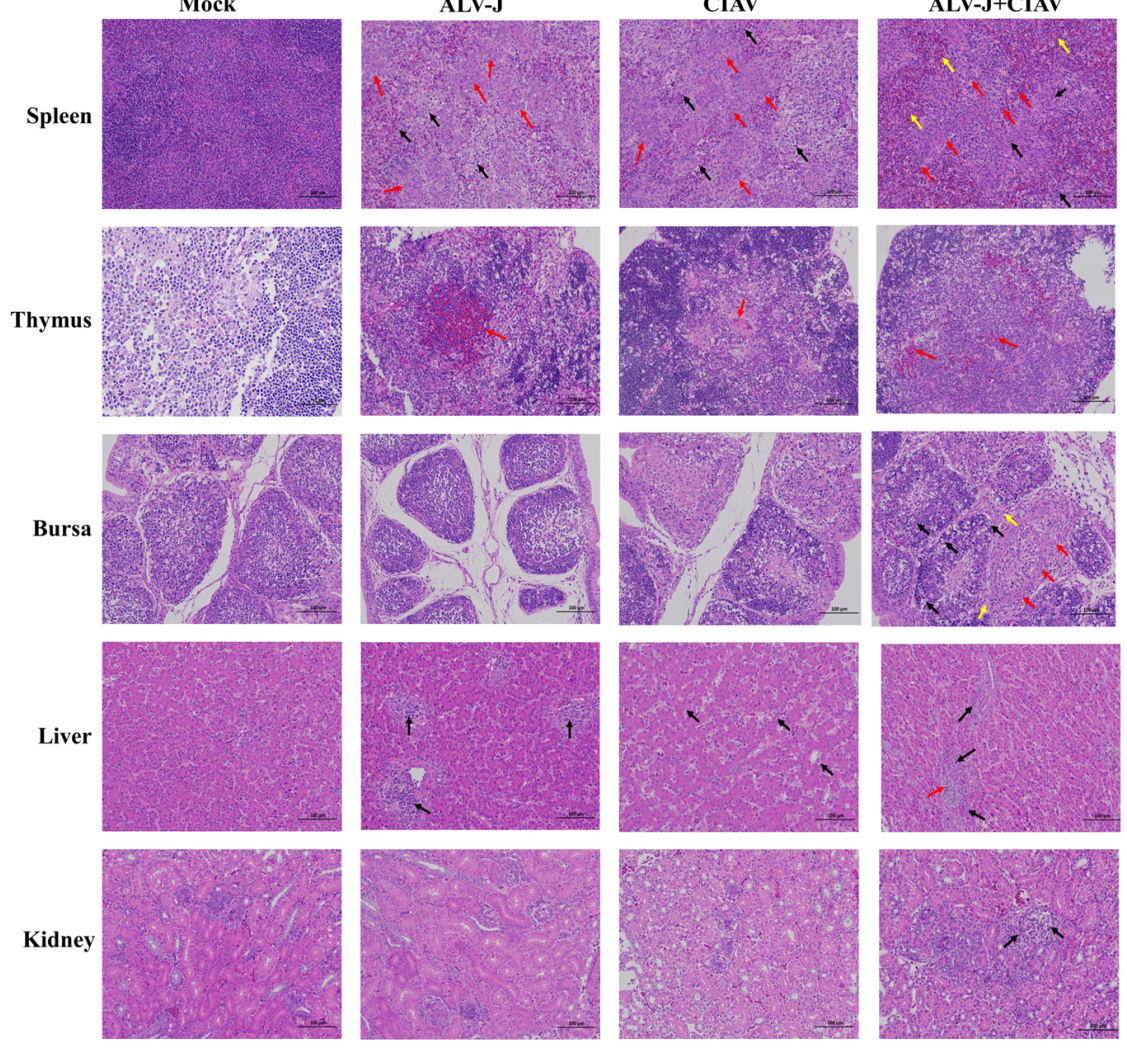
The histological examination of co-infection of ALV-J and CIAV. The thymus, Fabricius, spleen, liver, and kidney tissues were stained by HE (200×). The thymus tissue volume showed obvious atrophy, cortical thinning (black arrow), and necrosis of cells within the reticular structure occurred occasionally, along with cytoplasmic eosinophilic enhancement (red arrow). The Fabricius had an unclear boundary, with epithelial cells proliferation and a densely layered arrangement (black arrow), with a large amount of connective tissue hyperplasia in the follicular interstitium (red arrow) and inflammatory cell infiltration (yellow arrow). The spleen showed signs of lymphopenia (black arrow), reticulum cells proliferation (red arrow), and erythrocytoses (yellow arrow). The liver showed myeloma cells accumulation, l argernuclei, obvious eosinophilic granules in the cytoplasm (black arrow), and inflammatory cell infiltration (red arrow). The kidney exhibited inflammatory cell infiltration (black arrow).

To better understand the functional damage of organs caused by co-infection with ALV-J and CIAV, the spleen, thymus, Fabricius, liver, and kidney were collected and examined histologically. Compared to the ALV-J and CIAV mono-infected chickens, the histopathological observation results presented that ALV-J and CIAV co-infected chickens had the most severe lymphocyte loss in their immune organs, and more severe damage in liver and kidney manifested in more severe inflammatory cell infiltration (Figure 5). Taken together, these results clearly demonstrated that ALV-J and CIAV co-infection causes increased mortality, severe growth retardation, and tissue damage.

### 3.4. CIAV Can Promote the Replication of ALV-J In Vivo

**Figure 6:**
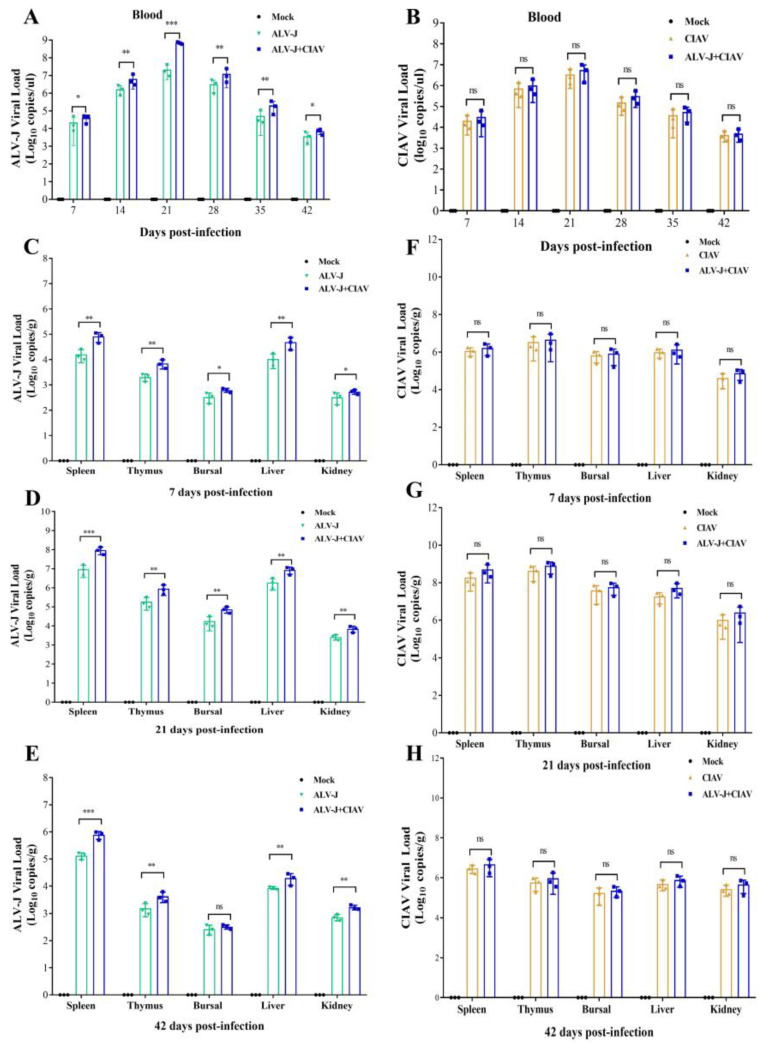
CIAV can promote the replication of ALV-J *in vivo*. (**A**). ALV-J viral load in blood of different groups of SPF chickens from 7 to 42 dpi. (**B**). CIAV viral load in blood of different groups of SPF chickens. (**C**–**E**). The viral load of ALV-J mono-infected groups and CIAV co-infected groups in the thymus, bursa, spleen, liver, and kidneys at 7 dpi, 21 dpi, and 42 dpi according to qPCR. (**F**–**H**). The viral load of CIAV mono-infected groups and ALV-J co-infected groups in thymus, bursa, spleen, liver, and kidneys at 7 dpi, 21 dpi and 42 dpi by qPCR. Data were expressed as mean ± SE and analyzed by One-way ANOV A Test. *, *p* < 0.05; **, *p* < 0.01; ***, *p* < 0.001; ns, no significant.

To further investigate whether ALV-J and CIAV could regulate the replication of each other *in vivo*, the viral loads of ALV-J and CIAV in venous blood and organs of infected chicken were detected using qPCR. The viral load of ALV-J in the venous blood of co-infected chickens was significantly higher than that of ALV-J mono-infected chickens at 7 dpi and 42 dpi, and this difference was extremely significant from 14 dpi to 35 dpi (Figure 6A). However, the viral load of CIAV in the venous blood of chickens in the co-infected group was not significantly different from that in the CIAV mono-infected group at all time points tested (Figure 6B). The viral load of ALV-J in the spleen, thymus, and liver from ALV-J and CIAV co-infected chickens was extremely significantly higher than that of ALV-J mono-infected chickens at 7 dpi, 21 dpi, and 42 dpi (Figure 6C–E). The ALV-J viral load in the Fabricius of co-infected chickens was significantly higher than that of ALV-J mono-infected chickens at 7 dpi, and this difference was extremely significant at 21 dpi, while this difference was not significant at 42 dpi. Additionally, the kidneys of co-infected chickens had significantly higher ALV-J viral loads than those of ALV-J mono-infected chickens at 7 dpi, and this difference was extremely significant at 21 dpi and 42 dpi (Figure 6C–E). However, the viral load of CIAV in the spleen, thymus, Fabricius, liver, and kidney of chickens in the co-infected group was not significantly different from that in the CIAV mono-infected group at 7 dpi, 21 dpi, and 42 dpi (Figure 6F–H). These results demonstrate that CIAV can promote the replication of ALV-J in chicken peripheral blood and major organs, but ALV-J has no significant effects on the replication of CIAV *in vivo*.

### 3.5. ALV-J and CIAV Synergistically Enhances the Immunosuppression in SPF Chickens

**Figure 7:**
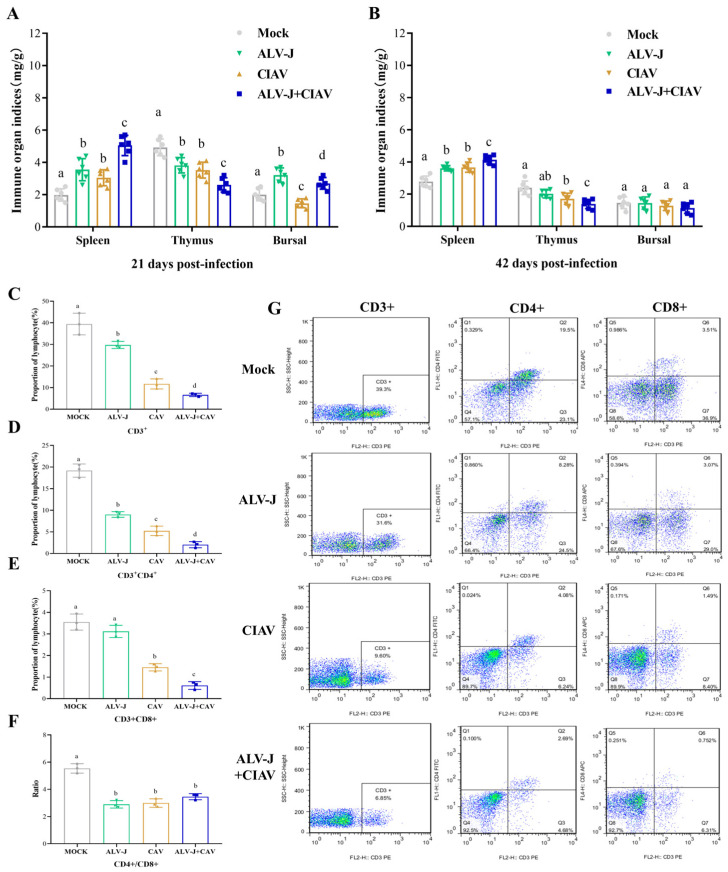
ALV-J and CIAV synergistically enhance the immunosuppression in SPF chickens. (**A**,**B**) Immune organ indexes of thymus spleen and bursa at 21 dpi and 42 dpi. (**C**–**F**) The proportion of lymphocyte subpopulation CD3^+^, CD3^+^CD4^+^, CD3^+^CD8^+^, and the ratio of CD4^+^/CD8^+^ are summarized in the diagram on the left. Data are expressed as mean ± SE and analyzed by a one-way ANOVA Test. Different lowercase letters indicate significant differences between different groups. (**G**) Representative FACS scatter diagrams showing the percentages of CD3^+^, CD4^+^, and CD8^+^ cells in peripheral blood mono-nuclear cells from treatment groups ALV-J, CIAV, ALV-J + CIAV, and empty control.

To determine whether co-infection of ALV-J and CIAV could synergistically promote more severe immunosuppression in chickens, we further evaluated the immune organ indexes and the differentiation of lymphocyte subsets. As described in Figure 7A,B, the spleen index was significantly higher in co-infected chickens than that in ALV-J or CIAV mono-infected chickens at 21 dpi and 42 dpi, indicating that co-infection can cause more serious splenomegaly. The thymus index was significantly lower in co-infected chickens than that in ALV-J or CIAV mono-infected chickens at 21 dpi and 42 dpi, indicating that co-infection caused more severe atrophy of the thymus gland (Figure 7A,B). At 21 dpi, the Fabricius index of ALV-J mono-infected chickens was significantly higher than that of co-infected chickens, CIAV mono-infected chickens, or uninfected chickens, and that of co-infected chickens was significantly higher than that of CIAV mono-infected chickens or uninfected chickens (Figure 7A). At 42 dpi, there was no significant difference in the Fabricius index among all groups (Figure 7B). Next, we measured the proportions of CD3^+^, CD4^+^, and CD8^+^ cells in the blood to assess the cellular immune responses after infection. Compared to the controls, the proportion of CD3^+^, CD3^+^CD4^+^, and CD3^+^CD8^+^ cells in the blood of co-infected chickens and mono-infected chickens significantly decreased. Furthermore, the proportion of CD3^+^, CD3^+^CD4^+^, and CD3^+^CD8^+^ cells in the blood of CIAV mono-infected chickens were significantly lower than that of ALV-J mono-infected chickens, while the proportion of CD3^+^, CD3^+^CD4^+^, and CD3^+^CD8^+^ cells in the blood of co-infected chickens was even more significantly lower than that of CIAV mono-infected chickens. Moreover, when compared to the controls, the ratio of CD4^+^/CD8^+^ in the blood of co-infected and mono-infected chickens was significantly lower. These data demonstrate that co-infection of ALV-J and CIAV can promote more severe immunosuppression in chickens than the single infection with either ALV-J or CIAV.

## 4. Discussion

ALV-J and CIAV co-infected chickens manifested greater disease severity and higher mortality than ALV-J or CIAV mono-infected chickens. However, the synergistic pathogenicity of ALV-J and CIAV in chicken is still incompletely elusive. Thus, a full comprehension of the synergistic pathogenic effects of ALV-J and CIAV co-infection is critical for providing efficient strategies to solve such production problems in the poultry industry.

### 4.1. Co-Infection of ALV-J and CIAV Can Significantly Aggravate the Severity of Illness In Vitro and In Vivo

Co-infection of viruses in chicken flocks is common in nature, and often alters the biological properties of viruses and cells, such as viral pathogenicity and cell apoptosis [31,32]. The outcome of any co-infection of viruses is complex and contributes significantly to disease severity and epidemiology. In this study, we established the cellular and animal co-infection model of ALV-J and CIAV to further understand the synergistic pathogenicity of ALV-J and CIAV *in vitro* and *in vivo*. Cytokines, such as IL-6, IL-10, IFN-α, and IFN-γ, have been shown to be involved in the progression of disease [16]. Thus, our study is concerned with the secretion of IL-6, IL-10, IFN-α, and IFN-γ in ALV-J and CIAV co-infected HD11 cells, which can be employed as target cells both for ALV-J and CIAV [14,33]. Consistent with previous studies [34,35], co-infected cells were found to have significantly higher levels of IL-6, IL-10, IFN-α, and IFN-γ at different time points (Figure 2), manifesting synergistically enhanced pathogenicity of both ALV-J and CIAV. Additionally, the apoptosis rate of the co-infected HD11cells was significantly higher than that of mono-infected or mock-infected cells at the different time points tested (Figure 3), revealing that ALV-J and CIAV can synergistically promote apoptosis and cause more serious harm to HD11 cells. Furthermore, we found that co-infection with ALV-J and CIAV significantly increased mortality and caused more severe growth retardation in chickens when compared to the ALV-J or CIAV mono-infection (Figure 4). Moreover, our histopathological examination revealed more serious tissue damage in the ALV-J and CIAV co-infected chickens (Figure 5). These results have consistently demonstrated that co-infection with ALV-J and CIAV could significantly aggravate the severity of illness *in vitro* and *in vivo*.

### 4.2. CIAV Can Efficiently Promote the Replication Ability of ALV-J

The most common outcome of co-infection is the effect of one virus on the replication of another, which is known as viral interference, whereby one virus can competitively constrain or promote the replication of the other virus within the host cell. Recent studies have shown that synergistic replication of ALV-J and MDV or ALV-J and REV is essential for boosting viral pathogenicity in chickens [17,19]. In the present study, we found that the ALV-J viral load was significantly higher in co-infected HD11 cells than in ALV-J mono-infected HD11 cells at all the detection time points (Figure 1B), indicating that CIAV can significantly promote ALV-J replication *in vitro*. However, the CIAV load in co-infected and CIAV mono-infected HD11 was not significantly different at any time detected at the stage of infection (Figure 1C), suggesting that ALV-J cannot enhance the CIAV replication *in vitro*. Notably, co-infected chickens had significantly higher ALV-J loads than ALV-J mono-infected chickens in the peripheral blood and in each organ at all the detection time points, which was consistent with the *in vitro* data and indicated that CIAV could significantly enhance ALV-J replication *in vivo*, while ALV-J still could not promote the replication of CIAV *in vivo*, according to the results of the CIAV viral load between the co-infected and CIAV mono-infected chickens. Various factors can influence viral synergistic interactions in co-infection, such as specific crosstalk between the two viruses and the induced immune response [36,37]. Viruses may hijack the host factors for their replication and meet the requirements associated with virus replication [38,39]. For example, miR-155 facilitates the synergistic replication between ALV-J and REV by targeting the PRKCI-MAPK8 and TIMP3-MMP2 dual-pathway [40]. Thus, it is reasonable to speculate that there is a competitive relationship between ALV-J and CIAV replication during the co-infection. The underlying reason for the interesting phenomenon that CIAV can enhance ALV-J replication *in vitro* and *in vivo*, but not vice versa, requires further investigation. Taking our findings together, the synergistic pathogenicity of ALV-J and CIAV co-infection revealed that CIAV can efficiently promote the replication ability of ALV-J, which may result in more serious immunosuppression in chickens. 

### 4.3. Co-Infection of ALV-J and CIAV Can Aggravate Immunosuppression

Previous studies have consistently confirmed that the severity of immunodeficiency increases when one or both immunosuppressive viruses infect the same host. Co-infection of ALV-J with REV, IBDV, or MDV in chickens can result in more severe immune organ damage and T and B lymphocyte exhaustion [17,19,41]. In our study, the spleen index was significantly higher in ALV-J and CIAV co-infected chickens than in mono-infected chickens, which intuitively reflected that co-infection can cause more serious splenomegaly. Furthermore, the decrease in the thymus index in ALV-J and CIAV co-infected chickens indicated that co-infection caused more severe atrophy of the thymus gland (Figure 7A,B). T cells play a pivotal role in the defense against viral invasions, immune surveillance, and antiviral immunity [42]. CD4^+^ T cell activation, following differentiation into Th1 and Th2 subsets, is involved in both cellular and humoral immunity [43]. CD8^+^ T cells are primarily induced by the apoptosis of target cells resulting from direct damage or viral infection, contributing to cellular immunity [44,45]. Changes in the proportion of CD4^+^ and CD8^+^ T cells directly impact the immune status of the organism. Our results showed that the proportion of CD3^+^, CD3^+^CD4^+^, and CD3^+^CD8^+^ cells in the blood of CIAV mono-infected chickens was significantly lower than that of ALV-J mono-infected chickens or uninfected chickens, indicating that CIAV induces severe lymphocyte apoptosis in peripheral blood. Furthermore, the proportions of lymphocytes exhibited more severe decreases in the blood of co-infected chickens compared to that of CIAV mono-infected chickens, revealing that ALV-J and CIAV co-infection may exacerbate immunosuppression.

In summary, this study demonstrates a synergy between ALV-J and CIAV in co-infected cells and chickens, as reflected by the fact that ALV-J and CIAV co-infection induced a more severe pathogenicity and worsened the immunosuppression of chickens. It provides a warning for the prevention and control of ALV-J and CIAV and the necessity of eradicating ALV-J or CIAV in large-scale chicken farms.

## Data Availability

All the data generated or analyzed during this study are included in this article. The datasets were deposited in a publicly accessible repository; the datasets generated for this study can be found in GenBank: https://www.ncbi.nlm.nih.gov/Genbank (accessed on 30 March 2024). Genbank accession numbers are mentioned in the Materials and Methods Section 2 of the article.
